# Are parental ADHD problems associated with a more severe clinical presentation and greater family adversity in children with ADHD?

**DOI:** 10.1007/s00787-013-0378-x

**Published:** 2013-02-06

**Authors:** Sharifah Shameem Agha, Stanley Zammit, Anita Thapar, Kate Langley

**Affiliations:** 1 Child and Adolescent Psychiatry Section, Institute of Psychological Medicine and Clinical Neurosciences, Cardiff University School of Medicine, Cardiff, UK; 2 Child Adolescent Mental Health Services Network, Cwm Taf Health Board, Cardiff, UK; 3 MRC Centre for Neuropsychiatric Genetics and Genomics, School of Medicine, Cardiff University, Cardiff, UK; 4 School of Social and Community Medicine, University of Bristol, Bristol, UK

**Keywords:** ADHD, Parental ADHD, Family environment

## Abstract

Although Attention Deficit Hyperactivity Disorder (ADHD) is recognised to be a familial and heritable disorder, little is known about the broader family characteristics of having a parent with ADHD problems. The main aim of this study was to investigate the relationship between parent ADHD problems, child clinical presentation and family functioning in a sample of children with ADHD. The sample consisted of 570 children with ADHD. Child psychopathology was assessed using a semi-structured diagnostic interview. Questionnaires were used to assess ADHD in the parents (childhood and current symptoms), family environment and mother/father-child relationship. Parental ADHD problems were associated with a range of adverse clinical outcomes in children with no difference in effects for mothers with ADHD problems compared to fathers with ADHD problems. Levels of maternal hostility were higher in families where mothers had ADHD problems, but reduced where fathers had ADHD problems. Parental ADHD problems index higher risk for more severe clinical presentation of ADHD in children and higher levels of family conflict (where there are maternal but not paternal ADHD problems). This study highlights that children with more severe behavioural symptoms are more likely to have a parent with persistent ADHD which has important implications when considering treatment and intervention strategies.

**Electronic supplementary material:**

The online version of this article (doi:10.1007/s00787-013-0378-x) contains supplementary material, which is available to authorized users.

## Introduction

It is well established that Attention Deficit Hyperactivity Disorder (ADHD) is a familial and highly heritable disorder [36]. Previous studies have also shown elevated rates of ADHD in the parents of children with ADHD and vice versa [10, 24].

Little is known about the relationship between parent ADHD, the severity of child ADHD and other clinical and family factors. There is increasing recognition of the significance of ADHD symptoms in adults. Adults with ADHD are reported to have much impairment in the form of repeated life failures such as academic underachievement, frequent job changes, marital breakdown and high rates of divorce [4, 41]. The impairments and difficulties faced by a parent with ADHD could impact on family functioning and the presentation of ADHD in their children.

The family environment is thought to be an important aspect in development, outcomes and manifestation of a disorder in children [20]. Previous literature shows that families of children with ADHD encounter greater difficulties such as family conflict, negative parent child relationship and higher rates of parent psychopathology [6, 8]. Parenting studies have linked parent ADHD to less effective parenting [5, 27]. High levels of maternal ADHD symptoms were found to interfere with improvement shown by children with ADHD following parent training [34]. It has also been found that one predictor of persistent ADHD in children was exposure to maternal psychopathology [9].

Parental psychopathology is clearly important, not only as an index of inherited risk but because of the role a parent plays in providing care and in becoming a role model to the child. Having a parent with ADHD may index additional risk to the child, influencing the course or persistence of ADHD in the child. Unfortunately, few studies have investigated the relationship between parent ADHD and child’s clinical presentation and findings have so far been inconsistent. This difference could be due to different definitions and timing of parental ADHD used in each study. Parent ADHD has been either measured only during childhood or only currently in adult life [16, 35]. In a study of siblings of ADHD and control probands, offspring were categorised into groups based on presence of parental ADHD before and after the birth of the child, but not necessarily concurrent to child ADHD assessment [7].

To diagnose ADHD in adults, clinicians often use the symptom criteria outlined in the DSM-IV as a guideline. One of the requirements is to establish the presence of symptoms both during childhood and 6 months before interview [38]. This definition of ‘Adult’ ADHD will be used in this study. Given the inconsistency of results and definitions of parent ADHD, it would be useful to explore how differences in the timing of parental ADHD relate to child and family functioning specifically by comparing persistent parental ADHD (‘adult’ ADHD criterion) with remitted ADHD (symptom criteria only met during childhood).

Understanding the influence of parent ADHD has important clinical relevance; if having a parent with ADHD indexes a more severe child clinical presentation, regardless of whether these links are inherited and/or environmental, then it may be important to ask about parental history during clinical assessment.

Literature on familial models has suggested gender differences in prevalence of ADHD exist due to the different burden of risk in males and females. It is suggested that females with ADHD require a greater load of risk than males before manifesting symptoms [11, 14]. By extending this into adulthood, literature suggests that females transmit a greater risk to their offspring than affected males [16, 31]. Thus a mother with ADHD may convey a greater risk to her offspring compared to a father with ADHD. Evidence reveals mixed findings regarding different risk effects of parental gender. Biederman and colleagues [7] found no significant differences in effect of parent gender on clinical outcome and family functioning. In another study [16], maternal ADHD was found to have greater influence on child impairment but on the other hand, Takeda and colleagues [35] found paternal ADHD to have a greater influence instead. Therefore, it may be important to clarify if there are differences of having a mother or father with ADHD.

This study aims to investigate the association between parent ADHD and child clinical presentation in a UK sample of children with ADHD and examine if there are any differences according to which parent has ADHD. It was hypothesised that children with a parent with ADHD problems will have a more severe clinical presentation of the disorder compared to children without a parent with ADHD and that there will be less warmth and higher hostility in this subgroup of children.

## Methods

### Sample

The sample of children with ADHD and their parents was obtained from a study of ADHD (Study of ADHD Genes and Environment, SAGE). Participants were recruited from child and adolescent psychiatry and paediatric services in the UK. All children referred had a clinical diagnosis of ADHD or were currently being assessed for a diagnosis. Each child had to be living with one biological parent. All children were of British Caucasian origin and met research diagnostic criteria for ADHD confirmed by research diagnostic interviews [1]. For these analyses, where more than one child from the same family participated in the study, one child was randomly selected. Parents and children gave informed written consent and assent before taking part in the study. Ethical approval for the study was obtained from the Wales Multicentre Research Ethics Committee.

### Measures

*Parent psychopathology.* Mothers and fathers each completed a questionnaire regarding the presence of ADHD symptoms in themselves at age 7–11 years (childhood) and in the last 6 months (current), using an 18-item checklist of DSM-IV ADHD symptoms [3]. Cronbach’s alpha reliability for parental ADHD measures ranged from 0.91 to 0.94. Symptom presence was rated on a Likert scale from 0 to 3 (‘not at all’, ‘just a little’, ‘pretty much’ and ‘very much’). Ratings of ‘pretty much’ or ‘very much’ were taken to indicate the presence of a symptom. Total scores were generated for childhood and current symptoms separately. Positive ADHD status was assigned if symptom criteria were met for any DSM-IV ADHD subtype (e.g. 6 inattentive symptoms, 6 hyperactive/impulsive symptoms or both). Our primary ADHD measure, ‘Adult’ ADHD reflected DSM-IV symptom criteria met in both childhood and current reports. In further analyses, a measure of ADHD in childhood-only was generated for those who met ADHD status in childhood, but not currently; 546 mothers and 280 fathers had provided data on their ADHD symptoms. The sample consisted of many single parent families; 58.7 % (mostly mothers) of which many had fathers without questionnaire data available (50.9 %).

*Child psychopathology.* Child psychopathology was assessed using the Child and Adolescent Psychiatric Assessment (CAPA), a semi-structured research diagnostic interview [1]. The parent CAPA was used to assess the child’s clinical symptoms of ADHD, oppositional defiant disorder, conduct disorder (CD), tic disorder, anxiety disorder and depression. The child version of the CAPA (excluding ADHD section) was additionally used for children aged 12 years and above [2]. All interviews were administered by trained psychologists supervised weekly by a child psychiatrist (A.T.). Symptom scores and diagnosis were generated from the CAPA according to DSM-IV criteria. To assess pervasiveness of ADHD symptoms, reports from schools were obtained using the Child ADHD Teacher Telephone Interview (ChATTI) [18], the Conner’s Teacher Rating Scale or DuPaul teacher rating scales [12, 13]. Conduct, anxiety and depression symptoms were counted as present when endorsed by either the parent or child.

Impairment of ADHD symptoms on functioning in several areas of life was assessed (e.g. home, school, with peers). Parents were asked to rate their child’s ADHD symptoms as interfering ‘never’, rarely’, ‘sometimes’ or ‘often’. Impairment scores of ‘sometimes’ or ‘often’ were taken as indicating significant impairment. Based on this, a total summed impairment score out of 8 was obtained. Cognitive ability was assessed using the Wechsler Intelligence Scale for Children IV (WISC-IV) [39]. From this, measures of full scale IQ and working memory index scores were obtained.

*Family factors.* Parents completed questionnaires rating the family environment and parent warmth and hostility at home. Two subscales from The Family Environment Scale (FES) were used to assess family environment; nine items on family conflict and nine items on family cohesion. [25]. The items were rated on a Likert scale from 1 to 4 (strongly disagree to strongly agree).

Parent warmth and hostility was assessed using the Iowa Youth and Families Project Interaction Ratings Scales [23] that comprises six items of warmth and four items of hostility rated on a Likert scale of 1 to 7. Children aged 12 years and above also rated their relationship with their mothers and fathers separately. This included five items of warmth and five items of hostility [23]. All family environment measures were reverse coded to reflect negative outcomes; higher scores reflected high conflict and hostility and low cohesion and warmth.

Information on demographics, family background and history of mental health was obtained from each family. Families were categorised as having a lower social economic status or not according to classifications from the Standard Occupational Classification. Information on medication was also collected and children were classified according to whether or not they had a current prescription for ADHD medication.

### Analysis

Analyses were conducted using linear regression for continuous outcomes and binary logistic regression for categorical outcomes. For variables that were not normally distributed, scores were transformed using the square root and natural logarithmic transformations. Child age and gender were used as covariates.

To investigate the relationship between parent ADHD and child clinical presentation, data were analysed using the ‘Adult’ measure of parental ADHD for mothers and fathers separately. Further analysis was conducted to explore differences in timing of parental ADHD by looking at mother and father ADHD status in childhood-only. Direct comparisons between mother and father ADHD groups as well as between the ‘Adult’ ADHD group and parent ADHD in childhood-only group were also conducted.

## Results

The sample consisted of 570 children, 88 (15.4 %) females and 482 (84.6 %) males, with a mean age of 10.78 years (SD 3.01 years). This male to female ratio here is typical of a clinical ADHD sample [15]. Approximately 73 % of children had a diagnosis of ADHD DSM-IV Combined type, 6 % with DSM-IV inattentive subtype, 8 % with DSM-IV hyperactive-impulsive subtype and 13 % ADHD DSM-III-R (children were classified as DSM-III-R where teacher reports were unobtainable but evidence of pervasiveness was present from parent reports). Rates of comorbid disorders were 40.2 % for oppositional defiant disorder, 17.8 % for CD, 7.5 % for any anxiety disorder and 0.8 % for any depression diagnosis.

High rates of parental ADHD problems were found in this sample; 29 % of parents met criteria for ‘adult’ definition of ADHD (See Table 1). The rates of ADHD in fathers are quite high despite there being many missing fathers. There also seems to be little overlap where both parents have ADHD.

**Table 1:** ADHD problems in mothers and fathers

Parent ADHD status	‘Adult’ ADHD	No ‘Adult’ ADHD	ADHD childhood only
Mother	102 (18.8 %)	441 (81.2 %)	54 (10.1 %)
Father	70 (25.3 %)	207 (74.7 %)	68 (25.2 %)
Either parent^a^	164 (28.9 %)	404 (71.1 %)	115 (20.5 %)
Both mother and father^b^	8 (1.4 %)	560 (98.6 %)	7 (1.2 %)

^a^either mother or father with an ‘Adult’ ADHD status

^b^Both mother and father with an ‘Adult’ ADHD status

Child age and gender did not significantly differ between those with or without a parent with ADHD. Both maternal and paternal ‘Adult’ ADHD were significantly associated with lower social class. There were no differences in the percentage of children taking stimulant medication for ADHD in the different parent ADHD groups.

### Child clinical presentation

Maternal ‘Adult’ ADHD was associated with greater child total ADHD and inattention symptom severity. Children with mothers in the ‘Adult’ ADHD group were more likely to have a diagnosis of CD (OR 1.79, 95 % CI 1.06–3.02, *p* < 0.05) and increased conduct symptom severity (See Table 2).

**Table 2:** Linear and Logistic Regressions—Mother ‘Adult’ ADHD and Child clinical presentation

Mother ‘Adult’ ADHD					
Child clinical presentation	No ADHD (*n* = 441)	ADHD (*n* = 102)			
	Mean (SD)	Mean (SD)	*β*	95 % CI	*p*
ADHD severity	15.05 (2.77)	15.62 (2.42)	0.09	(0.004, 0.28,)	0.044
Inattention severity	7.42 (1.70)	7.75 (1.66)	0.09	(0.001, 0.22)	0.048
Hyperactive-impulsive severity	7.63 (1.65)	7.87 (1.51)	0.06	(−0.03, 0.17)	0.15
ADHD impairment	6.70 (1.56)	6.99 (1.37)	0.07	(−0.01, 0.19)	0.08
CD symptom severity	1.17 (1.64)	1.59 (2.06)	0.09	(0.01, 0.22)	0.04
Oppositional defiant disorder severity	3.66 (2.32)	4.00 (2.27)	0.06	(−0.17, 0.82)	0.19
Depression symptoms	1.47 (1.47)	1.68 (1.51)	0.06	(−0.03, 0.16)	0.15
Anxiety symptoms	0.88 (1.70)	1.06 (1.73)	0.05	(−0.06, 0.22)	0.26
Full scale IQ	82.59 (13.43)	82.02 (14.10)	−0.02	(−3.59, 2.42)	0.70
Working memory	84.79 (14.46)	83.16 (12.58)	−0.04	(−4.76, 1.60)	0.33

	*n* (%)	*n* (%)	OR	95 % CI	*p*
ADHD DSM-IV combined	305 (72.1)	76 (78.4)	1.40	(0.81, 2.40)	0.22
ADHD DSM-IV inattentive	26 (6.0)	5 (4.9)	0.77	(0.28, 2.09)	0.60
ADHD DSM-IV hyperactive-impulsive	37 (8.5)	9 (8.8)	1.02	(0.47, 2.18)	0.97
CD diagnosis	68 (15.6)	25 (24.8)	1.79	(1.06, 3.02)	0.029
Oppositional defiant disorder diagnosis	180 (41.4)	38 (37.6)	0.84	(0.54, 1.32)	0.45

Paternal ‘Adult’ ADHD was associated with children’s total conduct symptom scores and there was weak evidence that children in the paternal ADHD group had a higher likelihood of having a diagnosis of CD (See Table 3).

**Table 3:** Linear and Logistic Regressions—Father ‘Adult’ ADHD and Child clinical presentation

Father ‘Adult’ ADHD					
Child clinical presentation	No ADHD (*n* = 207)	ADHD (*n* = 70)			
	Mean (SD)	Mean (SD)	*β*	95 % CI	*p*
ADHD severity	14.75 (2.62)	14.97 (3.38)	0.04	(−0.12, 0.24)	0.50
Inattention severity	7.41 (1.61)	7.35 (1.87)	−0.02	(−0.15, 0.12)	0.81
Hyperactive-impulsive severity	7.34 (1.71)	7.62 (2.11)	0.08	(−0.04, 0.24)	0.17
ADHD impairment	6.68 (1.66)	6.66 (1.38)	−0.04	(−0.17, 0.09)	0.55
CD symptom severity	0.98 (1.49)	1.53 (2.13)	0.14	(0.02, 0.29)	0.026
Oppositional defiant disorder severity	3.50 (2.37)	3.73 (2.44)	0.03	(−0.46, 0.84)	0.57
Depression symptoms	1.32 (1.24)	1.44 (1.31)	0.05	(−0.06, 0.16)	0.37
Anxiety symptoms	1.01 (1.87)	0.79 (1.40)	−0.03	(−0.23, 0.13)	0.58
Full scale IQ	84.21 (13.14)	80.20 (14.39)	−0.14	(−7.95, −0.43)	0.029
Working memory	85.60 (14.12)	81.17 (14.01)	−0.14	(−8,38, −0.47)	0.029

	*n* (%)	*n* (%)	OR	95 % CI	*p*
ADHD DSM-IV combined	131 (66.8)	47 (69.1)	1.02	(0.55, 1.90)	0.96
ADHD DSM-IV inattentive	20 (9.8)	3 (4.3)	0.45	(0.13, 1.59)	0.21
ADHD DSM-IV hyperactive-impulsive	20 (9.8)	4 (5.9)	0.61	(0.20, 1.86)	0.39
CD diagnosis	28 (13.7)	16 (22.9)	1.85	(0.93, 3.69)	0.08
Oppositional defiant disorder diagnosis	75 (36.8)	25 (35.7)	0.93	(0.53, 1.64)	0.80

### Family environment

Higher levels of conflict and lower levels of cohesion were reported by families in the maternal ADHD group, with weaker evidence of higher levels of mother hostility reported by children in these families (See Table 4).

**Table 4:** Linear regressions—mother and father ‘Adult ADHD and family environment

Mother ‘Adult’ ADHD					
Family environment	No ADHD (*n* = 441)	ADHD (*n* = 102)	*β*	95 % CI	*p*
	Mean (SD)	Mean (SD)			
Parent report: low warmth	10.82 (5.29)	11.47 (5.18)	0.06	(−0.04, 0.27)	0.16
Parent report: hostility	15.35 (4.37)	16.23 (4.67)	0.08	(−0.08, 1.85)	0.07
Child report: mother low warmth	11.32 (6.62)	12.61 (6.47)	0.09	(−0.08, 0.50)	0.16
Child report mother hostility	18.18 (6.63)	20.49 (6.69)	0.13	(−0.02, 4.60)	0.05
Child report father low warmth	14.87 (8.84)	17.93 (8.61)	0.13	(−0.61, 6.39)	0.11
Child report father hostility	17.59 (7.57)	19.52 (7.50)	0.94	(−1.22, 4.94)	0.24
Conflict	4.02 (2.36)	5.07 (2.37)	0.17	(0.54, 1.57)	<0.01
Low cohesion	2.18 (1.91)	2.68 (2.13)	0.10	(0.08, 0.94)	0.02

Father ‘Adult’ ADHD					
Family environment	No ADHD (*n* = 207)	ADHD (*n* = 70)	*β*	95 % CI	*p*
	Mean (SD)	Mean (SD)			
Parent report low warmth	11.93 (5.96)	10.12 (4.52)	−0.11	(−0.42, 0.00)	0.06
Parent report hostility	15.34 (4.40)	15.32 (4.81)	0.00	(−1.26, 1.29)	0.98
Child report mother low warmth	13.11 (7.68)	8.96 (4.20)	−0.22	(−0.90, −0.07)	0.02
Child report mother hostility	18.39 (7.02)	15.36 (7.33)	−0.17	(−6.18, 0.42)	0.09
Child report father low warmth	14.45 (8.87)	13.48 (7.68)	−0.02	(−4.23, 3.59)	0.87
Child report father hostility	18.52 (7.47)	17.56 (7.86)	−0.03	(−4.07, 2.98)	0.76
Conflict	4.11 (2.42)	4.20 (2.46)	0.02	(−0.59, 0.79)	0.78
Low cohesion	2.16 (1.86)	2.40 (2.04)	0.05	(−0.30, 0.78)	0.38

Children reported significantly higher levels of maternal warmth and weaker evidence of less mother hostility when fathers have ADHD (See Table 4). Contrary to what was found in the maternal group, there was no evidence of high levels of family conflict found in the paternal ADHD group. To further investigate this, we looked at these associations in subset of families where information was available from both parents (‘trios’ *n* = 96). Mother hostility was higher when mothers have ADHD compared to when fathers have ADHD (*p* < 0.01). (see supplementary Table 1).

### Parental ADHD in childhood-only

There was no evidence of differences in clinical presentation for children in the maternal childhood-only ADHD group. Children in the maternal childhood-only ADHD group had reported that mothers have less warmth. In the paternal childhood-only ADHD group, associations were found for lower hostility and better cohesion in the family. Mothers and fathers in these groups were not completely without current ADHD symptoms; few symptoms were present though not sufficient to meet symptom criteria for current diagnosis of any DSM-IV subtype [mean current ADHD symptoms: mothers 5.98 (2.69), fathers 4.46 (2.75).

Direct comparisons between the two groups show that children in the mother and father ‘Adult’ ADHD group have significantly higher symptom severity than those in the mother and father childhood-only group (results available from the first author).

### Further analysis with social class as covariate

As a separate analysis, we examined to what extent all observed associations changed after adjustment for social class. Adjusting for social class attenuated associations with child total ADHD symptoms, conduct symptoms, conduct disorder, and mother hostility by approximately 20–30 %. However, associations for inattention symptoms, family conflict, cohesion and maternal warmth were relatively unchanged (see Supplementary Table 2).

## Discussion

This is, to our knowledge, one of the first studies to investigate parent ADHD in a large clinical sample of children with ADHD in the UK. It includes the analysis of both parents and explores the timing of parent ADHD; persistent (‘Adult’ ADHD) and ADHD in childhood-only.

The prevalence of adult ADHD is estimated to be around 2.5–4.4 % [22, 33]. In this sample, we had found high rates of parental ADHD problems which were consistent to rates found in other studies of children with ADHD [16, 27]. Approximately a third of parents in this sample met criteria for the adult definition of ADHD (questionnaire assessed). This is noticeably high despite the stringent criterion set for the definition of adult ADHD.

The findings in this study suggest that having a parent with ADHD, particularly persistent/adult parental ADHD is associated with more severe clinical presentation in children with ADHD; maternal ADHD was associated with increased severity of overall ADHD, inattention and conduct symptoms and increased likelihood of CD in children. Paternal ADHD was found to be associated with the severity of children’s ADHD symptoms and a trend for higher rates of CD for children in the paternal ADHD group. The effect sizes of associations were relatively small apart from the prevalence of conduct disorder between those with and without a parent with ADHD. This may perhaps be due to less variability in the sample as all children have a diagnosis of ADHD. In other words, the association between parental ADHD and child characteristics might be stronger in general population (non-ADHD) samples.

Thus it appears that having a parent with persistent ADHD problems provides additional risk which is associated with a more severe clinical presentation of ADHD that could represent inherited or environmental risks as well as gene-environment interplay. Previous offspring twin studies suggest that the association between parent antisocial behaviour and child hyperactivity is genetic whilst the association to child conduct disturbance has genetic and environmental influences [32]. The transmission between parent ADHD and child problems, however, has not been explored beyond the investigation of inherited influences found in adoption studies. It is suggested that the effects of parent ADHD on child outcome could be transmitted through genetic effects as well as family environment through mechanisms such as parenting [21] but this requires investigation. Our study is not genetically informative and thus does not allow us to identify whether associations are inherited or environmentally mediated.

This study also investigated the differential effects of parent gender on offspring clinical presentation. There have been mixed findings on parent gender differences [7, 16, 35]. Our results seem to imply stronger evidence for influences of maternal ADHD. However, there were many missing fathers in our sample, and therefore power to find paternal effects was limited. Direct comparisons of mother and father ADHD in a subset of trios suggests that there are no differences on child clinical presentation between mother and father ADHD, though again power was limited.

We also investigated if parent ADHD was associated with adverse family environment. Maternal ADHD was associated with higher levels of conflict and lower levels of cohesion in the family. Although these are based on parent reports, there was a similar trend for higher levels of mother hostility reported by the children in this group. In a sample of affected sibling pairs, both maternal history of mood disorder and current ADHD was a predictor of impairment in family functioning [29].

One explanation for the effects of maternal ADHD on family environment could be because mothers are frequently the main caregiver and primarily responsible for the day-to-day organising for the family. Parenting a child with ADHD is already in itself challenging; parenting a child with ADHD when the parent has ADHD impairments themselves could be very stressful. This added stress may result in more conflict and hostility in the family. Parenting studies have found that parental ADHD symptoms were associated with decreased positive and involved parenting and more negative expressed emotion [17, 30]. Parental ADHD symptoms have also been found to be the strongest predictor of parental distress [37].

Interestingly, mothers were reported to be warmer to their children when fathers have ADHD. This may indicate that mothers living with a spouse with ADHD may be more empathic to their child’s ADHD symptoms. This is supported by findings from Minde and colleagues [24] who reported differences between perceptions of men and women who have a spouse with ADHD. Men appeared to be more critical and less tolerant if they were married to a woman with ADHD whereas, women were much more supportive and tolerant of husbands with ADHD. The differences in the effect of mother and father ADHD finds support from a study [19] which found that mothers with high ADHD symptoms offered more child blaming attributions when their child had ADHD whilst fathers with high ADHD symptoms offered fewer.

Low social class has previously been found to be associated with child mental health problems including ADHD. As low social class is also highly correlated with parent ADHD, we explored this by including social economic status as a covariate. Comparison of estimates showed that some associations were attenuated by 20–30 % and some remained relatively unchanged. However, it is not possible to distinguish whether social class is a confounder or acts as a mediator of the relationship between parent ADHD and child presentation and family functioning. For example, adults with persistent ADHD have functional impairments which lower their ability to achieve both educationally and occupationally. Thus, it is feasible that these individuals end up in a lower social class as a result of this. Consequently, growing up in this environment could increase the severity of ADHD in offspring (e.g. insufficient resources or support). Even if social class is a confounder the associations were not completely attenuated by adjustment for this. However, as for any observational study, we are unable to exclude the possibility of residual confounding for example by other characteristics associated with parent ADHD.

## Limitations

Firstly, this is a cross-sectional study; therefore we were unable to determine the direction of transmission from parent to child. Findings suggest that having a parent with persistent ADHD is associated with greater severity in children with ADHD. These findings are in need of replication as associations may not withstand correction for multiple testing. However, this was exploratory and findings add potential insight into how parent ADHD may be associated with presentation of ADHD in children. We found that having parents with only a childhood history of parent ADHD was not associated with more severe clinical presentation in children. This might suggest that exposure to parent ADHD behaviours during the child’s lifetime is more relevant. However, this needs to be explored further in studies with a genetically sensitive design.

Secondly, our definition of ‘Adult’ ADHD may be over restrictive therefore the percentage of parental ADHD may have been underestimated. Unfortunately there is no formal definition in diagnosing ADHD in adults. Given controversy and uncertainty in this area, it was decided that the diagnostic criteria in the DSM-IV was most reasonable to define adult ADHD. However, there was no measure of symptom impairment for parents in this study. Measures of parent ADHD were based on self report and retrospective measures for parent childhood ADHD. Nevertheless, evidence from previous studies has suggested that adults can give a reasonable account of their own childhood and current symptoms [26].

There may be possible shared rater bias as mostly mothers had rated child symptoms, family environment and parent child relationships. However, child reports of parent warmth and hostility showed similar directions of associations to parent reports. Teacher reports of child symptoms were available but a decision was made not to use these reports as 79 % of children in the sample were on medication for their ADHD, thus influencing child severity characteristics during school hours. In support of this, teacher rated ADHD severity was negatively correlated with child medication status.

Most families ascertained in this sample were not complete trios as there were many single parent families (mostly mothers). As a result of lack of information, the rate of father ADHD may have been underestimated. This limits power to examine whether paternal ADHD has more or less influence on child clinical presentation compared to maternal ADHD. However, by including data from single parent families makes this sample more representative of families with ADHD children. Significant differences were found between families of trios (both parents) and families with missing fathers similar to those reported by [40]. We were unable to examine the influences of parent ADHD separately by child gender as there were only a small number of girls, which is typical in clinically ascertained samples such as this.

### Clinical implications

This study highlights the importance of considering parent ADHD during clinical assessment. Results indicate that children with more severe behavioural symptoms are more likely to have a parent with persistent ADHD. Having a parent with ADHD problems could exacerbate or impede improvement in child symptoms through parenting and treatment administration. Screening parents during assessment of the child can help identify families with these problems, where parents may have more difficulties. It may be important to consider current treatment needs or interventions for the family as a whole. If further studies provide evidence that persistent parental ADHD is associated with the severity of child ADHD, this would encourage parenting programmes to cater for parents with ADHD with support and coping strategies for spouses. Perhaps treatment strategies can be extended to parents who have current symptoms as previous studies have found that treatment of other forms of parent psychopathology, notably depression might result in improvement in child symptoms [28].

## Conclusion

Overall, the results suggest children of parents with ADHD have more severe symptoms of the disorder compared to children without an affected parent. Family environment is also more adverse in these families especially when mothers have ADHD.

## Electronic supplementary material

Below is the link to the electronic supplementary material.

Supplementary material 1 (DOC 58 kb)
