# Supplementary material for: Are parental ADHD problems associated with a more severe clinical presentation and greater family adversity in children with ADHD?
Source: Eur Child Adolesc Psychiatry. 2013 Feb 6;22(6):369–77. doi: 10.1007/s00787-013-0378-x (PMC3669511; doi:10.1007/s00787-013-0378-x)
Supplement: Supplementary file 1 — Supplementary material 1 (DOC 58 kb) [file 787_2013_378_MOESM1_ESM.doc]

Supplementary Table 1

Means and comparison of parent ADHD group in trios n= 96

| Child report on parent warmth and hostility | Parent ADHD groups | | | |
| --- | --- | --- | --- | --- |
|  | No parent ADHD  (n = 60) | Mother ADHD only  (n = 12) | Father ADHD only (n = 21) | Both parents with ADHD  (n = 3) |
| Mother - low warmth | 12.41 (7.38) | 13.17 (7.37) | 9.05 (4.30) | 7.67 (4.62) |
| Mother - hostility | 18.26 (6.53) ^b^ | 20.85 (8.15) ^a^ | 13.57 (6.25) ^a, b^ | 23.67 (6.11) ^b^ |
| Father - low warmth | 14.03 (9.35) | 16.92 (7.89) | 12.57 (6.41) | 20.00 (15.00) |
| Father - Hostility | 18.61 (7.74) | 20.08 (7.05) | 16.38 (7.19) | 24.00 (11.53) |

Father ADHD only as comparison group

^a^ significant p<0.01

^b^ significant p<0.05

Supplementary Table 2

Comparison of estimates for associations unadjusted and adjusted for social class

|  | **Mother ADHD** | | | | | | | |
| --- | --- | --- | --- | --- | --- | --- | --- | --- |
|  | **Unadjusted for social class** | | | | **Adjusted for social class** | | | |
|  | *β * | | *p* | *95% CI* | *β  * | *p* | *95% CI* | |
| Total ADHD severity | 0.08 | | 0.07 | -0.01, 0.28 | 0.06 | 0.16 | -0.04, 0.25, | |
| Inattention severity | 0.08 | | 0.07 | -0.01, 0.22 | 0.08 | 0.07 | -0.01, 0.22 | |
| CD severity | 0.12 | | 0.01 | 0.03, 0.26 | 0.08 | 0.07 | -0.01, 0.22 | |
| Child report mother hostility | 0.15 | | 0.03 | 0.25, 5.15 | 0.12 | 0.09 | -0.30, 4.63 | |
| Conflict | 0.20 | | 0.00 | 0.68, 1.74 | 0.19 | 0.00 | 0.55, 1.62 | |
| Low Cohesion | 0.12 | | 0.01 | 0.17, 1.06 | 0.11 | 0.01 | 0.12, 1.01 | |
|  | *OR* | | *p* | *95% CI* | *OR* | *p* | *95% CI* | |
| CD Diagnosis | 2.01 | | 0.01 | 1.18, 3.44 | 1.70 | 0.06 | 0.98, 2.95 | |
|  | **Father ADHD** | | | | | | | |
|  | **Unadjusted for social class** | | | | **Adjusted for social class** | | | |
|  | *β* | *p* | | *95% CI* | *β* | *p* | | *95% CI* |
| CD severity | 0.12 | 0.07 | | -0.01, 0.27 | 0.09 | 0.17 | | -0.04, 0.23 |
| Child report mother low warmth | -0.20 | 0.04 | | -0.84, -0.02 | -0.20 | 0.04 | | -0.85, -0.01 |
| Full Scale IQ | -0.12 | 0.07 | | -7.62, 0.32 | -0.08 | 0.19 | | -6.52, 1.29 |
| Working memory | -0.13 | 0.05 | | -8.16, 0.07 | -0.10 | 0.12 | | -7.33, 0.88 |

* Unadjusted estimates do not match those in primary analysis (tables 2, 3 & 4) as unadjusted estimates here were conducted on the sample with no missing data on social class (n=515) to enable clear comparison with adjusted results. All associations were adjusted for age and gender.
